# Review of selected mosquito-borne diseases: arboviruses (dengue, chikungunya, Zika, West Nile, Japanese encephalitis, yellow fever) and parasitic diseases (malaria, lymphatic Filariasis)

**DOI:** 10.3389/fpubh.2025.1712094

**Published:** 2026-01-21

**Authors:** Yu-Lou Wei, Zhaoyu Wu, Run-Le Li, Feng Tang

**Affiliations:** 1 Research Center for High Altitude Medicine, Qinghai University, Xining, China; 2 Key Laboratory of the Ministry of High Altitude Medicine, Qinghai University, Xining, China; 3 Key Laboratory of Applied Fundamentals of High Altitude Medicine, (Qinghai-Utah Joint Key Laboratory of Plateau Medicine), Qinghai University, Xining, China; 4 Laboratory for High Altitude Medicine of Qinghai Province, Qinghai University, Xining, China; 5 Department of Health Management, Qinghai University Affiliated Hospital, Xining, China

**Keywords:** mosquito-borne diseases, transmission dynamics, global burden, vector control, vaccine, public health

## Abstract

Mosquito-borne diseases (MBDs) are a major global public health concern, accounting for 17% of infectious diseases and causing ~700,000 annual deaths. Transmitted by *Aedes*, *Anopheles*, and *Culex* mosquitoes, they include viral (dengue, Zika, chikungunya), parasitic (malaria, lymphatic filariasis), and zoonotic (Japanese encephalitis) pathogens. This review outlines key MBDs’ clinical features, global distribution—concentrated in tropics but expanding due to climate change and urbanization—and transmission dynamics shaped by environmental (temperature, humidity) and ecological (urban breeding sites) factors. It also summarizes control strategies: vector management, vaccines (e.g., R21 for malaria, IXCHIQ for chikungunya), chemoprophylaxis, and novel technologies. Moreover, persistent challenges are covered, which include insecticide resistance and socioeconomic costs (e.g., $318 billion for Aedes-borne diseases since 1975), emphasizing the need for integrated interventions.

## 1 Introduction

Mosquito-borne diseases (MBDs) comprise an important worldwide public health challenge, account for 17 % of all infectious diseases and cause approximately 700,000 deaths annually (1). The diseases are spread to humans and other vertebrates through the bite of infected mosquitoes, which act as biological vectors for a diverse range of pathogens, including viruses, bacteria and parasites. The intricate relationship between mosquito vectors, pathogens, and vertebrate hosts creates complex transmission dynamics that are highly sensitive to environmental, ecological, and socioeconomic factors.

At the core of MBD epidemiology is vectorial capacity, an index to measure the ability of a mosquito population to transmit pathogens. This capacity depends on several key factors: the number of mosquitoes, host-feeding behaviors, the development time of pathogens in the vectors (extrinsic incubation period), and survival rates of the vectors (2). Understanding these components is vitally important to develop effective control measures and predict outbreaks of the diseases.

Over 3,500 mosquito species have been identified, but only a small fraction act as disease vectors. The most medically important genera include *Aedes*, *Anopheles*, and *Culex*, which differ in habitat, breeding habits, and pathogen transmission capacity (3). It should be noted that the range of diseases transmitted by these three mosquito genera is far broader than those mentioned below. For instance, in addition to being the primary vectors of dengue, Zika virus disease, chikungunya fever, West Nile virus, and yellow fever virus, mosquitoes of the genus Aedes can also transmit diseases such as Rift Valley fever virus and Venezuelan equine encephalitis virus. Beyond transmitting filariasis and Japanese encephalitis, mosquitoes of the genus Culex are also one of the main vectors of West Nile virus. Although mosquitoes of the genus Anopheles are primarily characterized by transmitting malaria, some species may also be involved in the transmission of other parasites or viruses. The diseases listed in this article are only representative of those transmitted by each mosquito genus, not all types.

The spread of the chikungunya virus has been particularly severe in recent years. In Réunion alone, a French overseas department, it has caused more than 47,500 infections. Chikungunya virus has become one of the mosquito-borne viruses with the fastest spread and the widest coverage since 2025 (4). The most medically important genera consist of *Aedes*, *Anopheles*, and *Culex*. *An*opheles mosquitoes are primarily responsible for malaria transmission, while *Aedes aegypti* and *Aedes albopictus* are key vectors for dengue, Zika, chikungunya, and yellow fever viruses. Pathogens transmitted by *Culex* mosquitoes can contribute to filariasis, Japanese encephalitis, and other illnesses, among which Lymphatic filariasis invades body parts of the host,such as lymphatic system, subcutaneous tissue, abdominal cavity, chest cavity, and so on (5).

The severity of MBDs is unevenly distributed globally, with tropical and subtropical regions suffering heavily due to providing favorable climatic conditions for mosquitoes to grow, reproduce and spread diseases. However, global warming, globalization, and urbanization have expanded the geographic range of both vectors and diseases, leading to emerging threats in previously non-endemic areas (6). Facing this situation, it is urgent to have a comprehensive understanding of MBDs to support global health interventions, vaccine development and policies.

**Figure 1:**
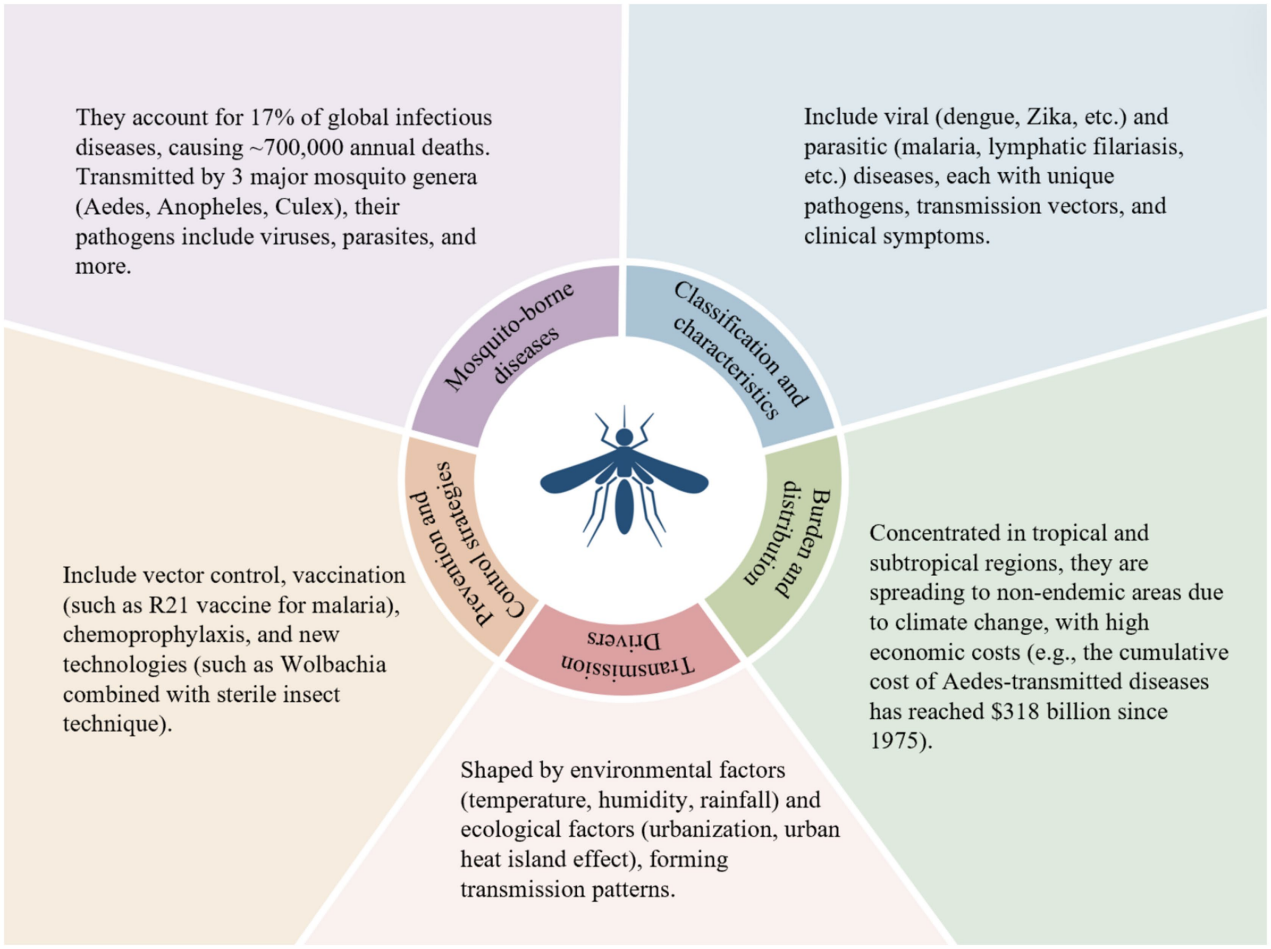
Systematically summarizes the key dimensions of mosquito-borne diseases: clarifies the disease classification (viral and parasitic), highlights the global disease burden (e.g., accounting for 17% of all infectious diseases and causing 700,000 deaths annually), elaborates on the main transmission drivers (environmental and ecological factors), and lists the core prevention and control strategies (vector control, vaccination, etc.). Interactions exist across various dimensions. For example, environmental factors (such as temperature and humidity) affect the survival of mosquito vectors and the replication of pathogens, thereby expanding the global distribution range of diseases.

Due to their high incidence rates, wide distribution, severe health hazards, and significant socioeconomic burdens, 6 types of arboviral diseases (dengue fever, chikungunya fever, Zika virus disease, West Nile virus disease, Japanese encephalitis, and yellow fever) and 2 types of mosquito-borne parasitic diseases (lymphatic filariasis and malaria) have become key focuses of global mosquito-borne disease prevention and control, and also the sole focus of this review (Figure 1).

## 2 Major mosquito-borne diseases: classification and clinical manifestations

### 2.1 Viral diseases

#### 2.1.1 Dengue fever

Dengue fever results from the dengue virus (DENV), a positive-sense single-stranded RNA virus that pertains to the *Flaviviridae* family, including 4 distinct types (DENV-1 to DENV-4), each of which can lead to disease from mildly febrile illnesses to severe ones,like dengue hemorrhagic fever (DHF) and dengue shock syndrome (DSS). This virus is mainly spread by *Aedes aegypti*—a vector highly adapted to urban artificial water-holding environments (e.g., construction site containers, flowerpots) (7).

Each year, dengue viruses transmitted by Aedes mosquitoes cause nearly 96 million symptomatic cases and 40,000 deaths. Meanwhile, there are over 3.9 billion people in 129 countries constantly facing the threat of dengue infection (8). Irregular vascular permeability is the main feature of DHF, which probably progresses to sudden hypovolemic shock—referred to as dengue shock syndrome (DSS)—brought on by severe plasma leakage. It is believed that a range of elements and mechanisms play a role in the development of DHF and DSS, including viral pathogenicity, antibody-dependent enhancement, immune dysfunction, host genetic predisposition, the nonstructural protein 1 (NS1) viral antigen, and anti-DENV NS1 antibodies (9).

There is a significant correlation between temperature changes and the transmission of dengue fever: 72.1% of dengue cases from 2010 to 2019 could be attributed to rising temperatures. The proportion of temperature-related cases and the incidence rate are higher during the period from the warm-dry season to the early rainy season (April–June), as well as in equatorial regions and areas with rapid urbanization. Temperature promotes dengue transmission through three main ways. First, it shortens the development cycle of Aedes mosquito larvae. Second, it extends the lifespan of adult mosquitoes. Third, within the temperature range of 26–30 °C, it increases the replication efficiency of the virus in mosquitoes and shortens the extrinsic incubation period (10).

Recent research: Dengue NS1 activates platelets via TLR4, possibly boosting aggregation, endothelial adhesion, macrophage phagocytosis, and causing higher vascular permeability, bleeding, thrombocytopenia. Also, anti-DENV NS1 antibodies may worsen severe dengue by prompting endothelial cells to release inflammatory mediators (MCP-1, IL-6, IL-8) (11).

Dengvaxia is intended for individuals with a prior history of dengue, while TAK-003 received approval from WHO in 2023 for administration to children aged 6–16 in highly endemic regions (12).

#### 2.1.2 Zika virus disease

Zika virus (ZIKV) is another flavivirus closely associated with mosquitoes of the *Aedes* genus, and its transmission mainly relies on this vector genus. Though Zika virus (ZIKV) was identified almost 70 years ago, it became a major health hazard in the 2010s. The latest epidemic in Latin America from 2015 to 2016 exposed a range of destructive effects of ZIKV infection, such as Guillain-Barré syndrome (GBS) in grown-ups and microcephaly caused by ZIKV in fetuses during the development period, leading to long-lasting cognitive problems in their later lives. Currently, ZIKV is endemic in numerous nations across Southeast Asia, Africa, and South America (13, 14). India has also recorded several historic local ZIKV outbreaks, sparking worries that a future ZIKV epidemic might be catastrophic in heavily populated regions of India and other countries (15).

The effective temperature range for Zika virus transmission is 18–34 °C, with the peak transmission efficiency occurring between 26 and 29 °C. When the temperature is below 18 °C or above 34 °C, the transmission probability and intensity decrease significantly to near zero (16).

Phylogenetic analysis: 2 major ZIKV lineages (African, with 1947 MR-766; Asian, linked to 2007 Yap/2013 French Polynesia/American outbreaks). Largely similar (≤5% amino acid difference); African strains more transmissible and pathogenic (17, 18). Although without approved vaccines for ZIKV, plenty of vaccine platforms/methods designed for other flavivirus uses have been used for ZIKV. While the research and development of ZIKV vaccine is still in the non-clinical stage,more than 60 institutions and firms are studying products to prevent the transmission of ZIKV (19).

#### 2.1.3 Chikungunya

Chikungunya fever arises from a virus spread by *Aedes aegypti* and *Aedes albopictus*, characterized by an incubation period of 1 to 12 days, and patients have typical acute-phase symptoms including high fever (often exceeding 39 °C), joint pain (a hallmark feature, mostly involving bilateral and symmetric joints such as the hands, feet, knees, and wrists), headache, fatigue, and muscle pain. Some patients may experience gastrointestinal symptoms and skin rashes (40–50%). It may also cause neurological complications (such as encephalitis, meningoencephalitis) and cardiovascular symptoms (such as arrhythmia, myocarditis). 40–80% of patients progress to the chronic phase, with symptoms lasting more than 3 months or even years, mainly characterized by chronic joint pain and inflammation. This phase is easily misdiagnosed as rheumatoid arthritis, and can also lead to reduced labor capacity and increased medical expenses (20, 21).

The disease is native to certain areas of Latin America and Asia, as well as a strip across central Africa reaching the Indian Ocean islands. In these regions, the warm and humid environment is particularly favorable to the mosquitoes transmitting the virus (22). Its global spread has caused severe local outbreaks: for example, in Réunion (a French overseas department), a single epidemic event led to more than 47,500 infections, highlighting the virus’s strong transmission capacity (4).

The relationship between Chikungunya transmission and temperature is not a simple “linear correlation” but a three-dimensional interaction of “temperature—viral replication—mosquito activity.” A fluctuating temperature range of 18–21 °C is the optimal interval for transmission; the virus can still replicate at low temperatures but mosquito activity is restricted, while transmission efficiency decreases at high temperatures (23).

During 1950s to 2009, there were 3 genotypes active,including West African genotype (West Africa only), Asian genotype which outbroke in Thailand in 1958 (phylogenetic Clusters A/early, B/recent) and ECSA with the widest distribution (central-southeastern Africa initially, global post-2004 3 subgroups) and 3 subgroups (Cluster III = key) of which IOL broke out in Indian Ocean during the year 2004–2009,and later spread to Asia, Europe and America where the virus mutated, contributing to mosquito infection (24).

Currently, two chikungunya virus (CHIKV) vaccines have recently been approved. The first one is a live-attenuated vaccine, VLA1553, named IXCHIQ; the other is a virus-like particle vaccine, PXVX0317 (25).

#### 2.1.4 West Nile fever

Globally, the primary vectors or enzootic vectors of West Nile virus (WNV) all belong to the genus *Culex* (e.g., Cx. modestus, Cx. pipiens, Cx. quinquefasciatus, Cx. tarsalis) (26). Most WNV infections are asymptomatic (accounting for 80%). Twenty percent of infected individuals develop West Nile fever, with symptoms including fever, headache, fatigue, myalgia, rash, and gastrointestinal symptoms. Less than 1% of infected individuals progress to neuroinvasive diseases (such as meningitis, encephalitis, and acute flaccid myelitis). Among these, acute flaccid myelitis is characterized by asymmetric limb weakness and may even lead to respiratory failure; cranial neuropathies (e.g., facial weakness) may also occur (27). Among older adult survivors of WNV- associated neuroinvasive diseases, up to 50% may experience post- illness sequelae, such as fatigue, dizziness, difficulty in concentrating, depression, anxiety, sleep disturbances, recurrent headaches, and even autoimmune diseases (27, 28). WNV was first discovered in Uganda in 1937 and detected in New York in 1999. Between 1999 and 2020, a total of 52,532 WNV infection cases were reported in the United States, including 2,456 deaths (26).

The optimal temperature range for WNV transmission is 23.8–25.2 °C, with slight variations depending on the species of *Culex* mosquito vectors. The minimum transmission temperature ranges from 8.0–19.0 °C, and the maximum from 31.8–34.9 °C. At low temperatures (below the minimum threshold), the extrinsic incubation period of WNV is prolonged and the activity and reproduction of *Culex* mosquitoes are inhibited. At high temperatures (above the maximum threshold), the lifespan of *Culex* mosquitoes is significantly shortened (a core limiting factor) and the virus infection rate is reduced; both scenarios block transmission. Within the optimal temperature range, the biting rate and lifespan of *Culex* mosquitoes, as well as the development efficiency of WNV, are synergistically optimized, reaching the peak of transmission potential (29).

Latest studies have confirmed that WNV has 9 genetic lineages, and the virulence varies among different lineages. The virulence characteristics of some lineages have been clarified, while further in-depth research is needed for the remaining lineages. Among them, Lineage 1 and *Koutango virus* (Lineage 7) exhibit relatively strong virulence; the virulence of Lineage 2 is controversial; the remaining lineages have relatively weak virulence or their virulence remains unclear (30).

Currently, there are no approved vaccines or treatments for WNV in humans. However, a variety of human vaccines have completed preclinical studies, and a few have entered Phase II clinical trials. Nevertheless, due to the sporadic nature of the epidemic and limited market attention, Phase III efficacy trials have not been conducted (31, 32).

#### 2.1.5 Yellow fever virus

Yellow fever virus (YFV) is a flavivirus spread by mosquitoes, mainly via the bite of *Aedes aegypti* to humans. Like other flaviviruses, fever, headache, muscle pain, nausea, and vomiting are main symptoms of it. However, its in-hospital case fatality rate (CFR) can significantly climb to 67%, a characteristic that makes the disease a major concern in the field of public health (33).

Key YFV high mortality debate: viral cytotoxicity (severe liver damage), excessive immune activation, or abnormal vascular endothelial function. Current research: abnormal endothelial function links to fatal outcomes, may mediate “viral toxicity-immune activation” (virus-induced factors like IL-6 disrupt junctions; damage worsens immune infiltration, vicious cycle) – more data needed (34).

The process of manufacturing the yellow fever vaccine is laborious and difficult to scale up rapidly, and these factors lead to persistent challenges in terms of vaccine availability. There are specific contraindications for the administration of the yellow fever vaccine (YF vaccine), mainly involving individuals with immunosuppression. As this vaccine is a live attenuated virus vaccine, extra caution is required when using it for such individuals; during outbreaks, whether to administer it to them must be evaluated on an individual basis (35, 36). Although effective vaccines such as YF-17D exist, the yellow fever virus still rages in tropical and subtropical regions, particularly the tropical areas of Africa and South America (37), with periodic outbreaks.

#### 2.1.6 Japanese encephalitis

Japanese encephalitis (JE) is an anthropozoonosis transmitted by mosquitoes, and it is classified as a viral zoonotic infection, as it is caused by the Japanese encephalitis virus (JEV) which belongs to the Orthoflavivirus genus. The pathogenic mechanism of JEV revolves around the axis of “immune regulation interference—blood–brain barrier (BBB) disruption—central nervous system (CNS) damage”: After entering the human body via mosquito bites, the virus first utilizes peripheral immune cells such as monocytes, macrophages, and dendritic cells (DCs) for replication and spread. Among these, JEV-infected DCs upregulate the expression of programmed death ligand 1 (PD-L1) to induce the expansion of regulatory T cells (Tregs), which suppresses the body’s antiviral immune response and helps the virus evade immune surveillance. Meanwhile, the virus stimulates brain microvascular endothelial cells (BMECs) to release high-mobility group box 1 protein (HMGB1), which impairs the integrity of the BBB and facilitates the entry of virus-carrying monocytes into the CNS through a “Trojan horse” mechanism. Additionally, exosomes secreted by JEV-infected cells carry viral RNA and proinflammatory mediators, further exacerbating the inflammatory response and neuronal apoptosis within the CNS, ultimately leading to neurological damage (38).

Most people infected with JE have mild symptoms or no symptoms at all, while a small number progress to severe encephalitis. The typical early symptoms include fever (mostly high fever), headache, nausea, and vomiting, with some patients also experiencing respiratory or gastrointestinal symptoms such as diarrhea and nasal congestion; as the condition progresses, disturbance of consciousness (e.g., drowsiness, irritability, coma), convulsions or seizures, and neck stiffness may occur, and tremors or spastic paralysis of the limbs can also appear, and in severe cases, the respiratory center is involved, leading to respiratory failure. Among patients who develop severe clinical illness (encephalitis), the mortality rate can be as high as 30% (39, 40).

JEV is primarily raging in 25 countries in Eastern and Southeastern Asia, containing rural areas of Japan, Thailand, the Philippines, and Indonesia. Pigs and ardeid birds, respectively, serve as the amplifying hosts and reservoir hosts of the virus. In some countries like China, human infection rates have declined due to vaccination programs and agricultural improvements. However, the transmission range of JEV continues to expand; for instance, it has spread to mainland Australia, where it has been detected in local mosquitoes, animals, and humans (40). The treatment of JE mainly focuses on symptomatic and supportive care, such as maintaining fluid and electrolyte balance and controlling high fever. Ribavirin or interferon may be used in the early stage of infection, while auxiliary methods like melatonin have not yet entered clinical practice. Existing JE vaccines include multiple types such as live attenuated vaccines, inactivated vaccines, and chimeric vaccines, which show good protection rates/durations and include type-specific vaccines. However, they have limitations including limited cross-protection, safety risks, high costs, and complex production processes. Experimental vaccines such as recombinant protein vaccines and DNA vaccines are mostly in the animal experiment stage, and their clinical applicability remains to be verified. Given the current situation of limited treatment options, shortcomings in existing vaccines, and unimplemented experimental vaccines, how to develop highly effective and low-cost vaccines more quickly has become the key to current JE prevention (41, 42).

### 2.2 Parasitic diseases

The representative types of mosquito-borne parasitic diseases mainly include malaria and lymphatic filariasis.

#### 2.2.1 Malaria

Malaria is a life-threatening parasitic disease caused by Plasmodium species, and *Plasmodium falciparum* is the major cause of the majority of severe cases and deaths. Transmitted exclusively by Anopheles mosquitoes, malaria remains one of the most fatal infectious diseases globally. According to a data of a report covering 83 endemic nations from WHO, there were 263 million cases recorded in 2023, showing a small rise from the 252 million cases in 2022. In the report, Sub-Saharan Africa stayed the most heavily impacted region by malaria, making up approximately 94% of all global cases in 2023. The countries bearing the greatest burden included Nigeria (30.9%), the Democratic Republic of the Congo (11.3%), Niger (5.9%), and the United Republic of Tanzania (4.3%) (43, 44).

Temperature plays a critical role in malaria transmission dynamics, affecting both mosquito development and parasite replication. Climate change models predict changes in the prevalence of malaria: some temperate regions will become more suitable for transmission, while some tropical regions may see a reduced risk due to temperatures exceeding the heat tolerance of the parasite (45, 46). Malaria parasites own a complicated life cycle, with sexual reproduction occurring in mosquito vectors and asexual replication taking place in human hepatocytes and red blood cells (43). The full sequencing of the genomes of multiple Plasmodium species has started a new stage in malaria research, offering novel approaches that speed up both the discovery of drugs and the creation of vaccines. Between 2016 and 2024, a number of nations earned WHO certification as malaria-free, such as Algeria and Argentina in 2019, China in 2021, Azerbaijan and Tajikistan in 2023, and Belize and Egypt in 2024—all of which demonstrate the effectiveness of malaria elimination efforts. 2024 witnessed another significant breakthrough when R21/Matrix-M malaria vaccine gained its approval from WHO for reaching the 75% efficacy goal among African children (47).

#### 2.2.2 Lymphatic Filariasis

Lymphatic Filariasis (LF) is a usually neglected tropical disease caused by nematodes. There are three species of pathogenic nematodes, named *Brugia malayi*, B. timori, and *Wuchereria bancrofti*. Among them, *Wuchereria bancrofti* (*Wuchereria bancrofti*) accounts for 90% of global lymphatic filariasis cases. It has the widest geographical distribution, covering Africa, Asia, and parts of Latin America, and is also the main pathogenic nematode in endemic countries such as India. *Brugia malayi* (*Brugia malayi*) causes the remaining 10% of global lymphatic filariasis cases, and is mainly prevalent in southern and eastern Asia. *Brugia timori* (*Brugia timori*) has the most limited geographical distribution, being endemic only on some islands of Indonesia. Its proportion of cases is relatively low, and both its virulence and epidemic intensity are weaker.

It imposes health, social, and economic burdens on over 120 million people worldwide. *Wolbachia* provides essential metabolites (such as heme and riboflavin) for filarial worms, while filarial worms offer a living environment for *Wolbachia*. This symbiosis is a prerequisite for subsequent pathogenesis. Moreover, “the host’s inflammatory response to filarial worms and possibly to the symbiotic bacterium *Wolbachia* is the cause of lymphatic damage and disease onset.” After *Wolbachia* is released, its surface proteins (e.g., WSP) and lipopolysaccharides (LPS) are recognized by the host’s immune system, which activates receptors like TLR-2 and TLR-4 and triggers the release of pro-inflammatory cytokines (such as TNF-*α* and IL-6),without treatment, it can lead to elephantiasis, lymphedema, hydrocele, etc. (48–50).

Among 81 endemic countries, 53 have implemented Mass Drug Administration (MDA). From 2000 to 2009, over 2.8 billion drug doses were distributed to 845 million people worldwide to prevent the transmission of lymphatic filariasis, a parasitic disease. Currently, the core of lymphatic filariasis treatment is MDA (Mass Drug Administration). IDA (Ivermectin, Diethylcarbamazine and Albendazole triple therapy) and doxycycline address the partial needs of “effectively killing microfilariae” and “killing adult worms” respectively, yet they still face key challenges including “lack of broad—spectrum adulticidal drugs, high R&D risks, and incomplete application coverage.” To achieve the WHO’s 2030 elimination target, it is necessary to accelerate the R&D of new drugs (especially pediatric formulations), optimize treatment strategies, and advance vaccine research (48, 51).

## 3 Global disease burden and distribution

### 3.1 Spatiotemporal distribution

The epidemic areas of dengue fever have gradually expanded from local concentration to global spread. Historically, dengue virus infection was first reported in 1780, and caused large-scale concurrent epidemics in port cities of Asia, Africa, and North America in the late 18th century. The number of cases surged during World War II, then decreased due to the prevention and control of *Aedes aegypti*, but re-spread significantly after 1980. A study on 76 countries around the Indian Ocean from 1952 to 2009 showed that dengue outbreaks occurred throughout the entire period in East Asia and Southeast Asia; South Asia and Central Asia had continuous outbreaks in phases; only a few countries in Africa had records of outbreaks lasting 4 years or more. In 1990, the global number of dengue cases was 30.668 million, with the core epidemic areas concentrated in South Asia and Southeast Asia. Local transmission of dengue was rare in high-income regions, where most cases were imported. In modern times, the global number of dengue infections has continued to increase since 2009, and at present, at least 100 countries worldwide have endemic transmission of dengue. By 2019, the global number of dengue cases had increased to 56.879 million. Southeast Asia and South Asia still bore the heaviest burden of dengue. East Asia had the fastest growth in age-standardized incidence rate, while Tropical Latin America ranked first in age-standardized death rate and age-standardized disability-adjusted life years (DALYs) rate. Some Pacific islands as well as countries in Asia and Latin America had high dengue incidence rates. The burden of dengue in high-income regions also increased significantly, with more reported cases of local transmission (52, 53).

From 1990 to 2021, there was an upward trend in the age-standardized incidence rate, prevalence rate, mortality rate, and disability-adjusted life year (DALY) rate of dengue among adults from 20 to 49 years old worldwide, with an annual growth rate exceeding 2%. It is said that by 2030, the age-standardized incidence and mortality rate of dengue among adults aged 20–49 worldwide are anticipated to reach 872.94 cases per 100 thousand people and 0.24 deaths per 100 thousand people, respectively (54). Driven by factors including global warming and increased human mobility, the distribution range of dengue fever has continued to expand, forming a pattern characterized by “traditional regions under pressure, emerging regions with growing burden, and non-endemic regions facing localized epidemics.”

The geographical distribution of malaria burden from 2000 to 2022 showed significant regional differences and dynamic evolution. On a global scale, sub-Saharan Africa has always been the core high-burden region for *Plasmodium falciparum* malaria. The infection prevalence and case incidence rate in this region have stagnated since 2015. Since 2015, the malaria mortality rate in the WHO African Region has decreased by 16%. However, the mortality rate of 52.4 per 100,000 at-risk populations in 2023 still remains far higher than the target of 23 deaths per 100,000 population set out in the Global Technical Strategy for Malaria 2016–2030 (55). Outside Africa, however, continuous progress was previously made in reducing the incidence rates of both *Plasmodium falciparum* and *Plasmodium vivax*. Only in 2022, the *Plasmodium vivax* epidemic caused by floods in Pakistan reversed the local improvement trend, becoming a key factor for the global increase in *Plasmodium vivax* cases (56).

Further machine learning analysis of data from 106 countries over the same period confirmed that the current malaria burden is highly concentrated in specific regions. Nigeria has the highest malaria incidence rate, while South Sudan, Zambia, and the Central African Republic rank first in mortality rate. Countries such as Benin, Burkina Faso, and Ghana have also formed geographical clusters with both high incidence and high mortality rates. The distribution of these clusters is closely related to socioeconomic factors such as basic health infrastructure and population growth (56, 57). Therefore, at the global level, it is necessary to focus on breaking the “prevention and control stagnation” dilemma in sub-Saharan Africa and increase investment in innovative prevention and control tools for this region. Meanwhile, a linked monitoring mechanism between climate disasters and malaria epidemics should be established to prevent epidemic resurgence. Furthermore, the application of technologies such as machine learning in burden prediction and cluster identification should be strengthened, which will provide important technical support for further accurately targeting key prevention and control areas and improving prevention and control efficiency.

The geographical pattern and disease burden of Zika virus have undergone a fundamental shift over the past few decades. Before 2015, its epidemic regions were highly limited and sporadic. First identified in Uganda in 1947, the virus circulated for a long time only in tropical areas of Africa (such as Uganda, Nigeria, Senegal) and Southeast Asia (such as Malaysia, Indonesia, Cambodia). Although outbreaks occurred in Yap Island of the Pacific in 2007 and French Polynesia from 2013 to 2014, the number of recorded human infections worldwide was extremely small (only 14 cases before 2007), and no sustained large-scale transmission occurred. The disease burden was mainly characterized by mild and asymptomatic infections (58). After 2015, the virus entered a global spread phase, with significant changes in its geographical distribution and burden characteristics. Originating primarily in Brazil, the American sublineage evolved from the Asian lineage rapidly spread across more than 30 countries and regions in the Americas, triggering a large-scale epidemic from 2015 to 2016. This marked the first clear association of Zika virus with congenital Zika syndrome and Guillain-Barré syndrome, elevating the disease burden to a severe public health crisis. In 2021, only 5 Global Burden of Disease regions reported cases of Zika virus infection, which were mainly centered in Latin America and the Caribbean, and most of the outbreaks occurred in areas with low-medium and medium sociodemographic indices (59, 60). As of 2023, local transmission has been reported in 92 countries and regions. While the Americas remain the core region with the highest number of cases, new epidemic hotspots have emerged, including India (with peak cases in 2018 and 2021, concentrated in Uttar Pradesh, Madhya Pradesh, etc.), Singapore, and Thailand in Asia, as well as Guinea and Mali in Africa. Even non-traditional epidemic regions such as Europe and the United States have frequently reported imported cases and potential transmission risks (60, 61).

In the past, the epidemic range of chikungunya fever was relatively narrow. After the virus was first isolated in what is now Tanzania in 1952, it was mainly transmitted through the sylvatic cycle formed by Aedes forest mosquitoes and non-human primates in sub-Saharan Africa. Meanwhile, cases were reported in parts of tropical regions such as Southeast Asia, the Indian subcontinent, and the Pacific. However, due to the limited distribution of major vectors like *Aedes aegypti* and *Aedes albopictus* and its reliance on specific sylvatic transmission cycles, its spread to other regions was relatively limited.2004 marked a key turning point for the expansion of its epidemic area, after which the transmission range of the virus widened significantly. Since 2004, chikungunya virus has been reported in more than 104 countries across Africa, Asia, the Americas, Europe, and Oceania. Among these, India bears the heaviest burden of the epidemic, with approximately 9.1 million cases annually. As *Aedes albopictus* entered Europe, local indigenous transmission and epidemic outbreaks also occurred in Italy and France. After 2013, cases have covered 115 countries, and major epidemics have occurred in most (sub)tropical regions of Central and South America. Recent data from the Pan American Health Organization (PAHO) shows that the number of reported cases in the Americas increased significantly by over 113,000 within 3 months, including 51 deaths. At the same time, virus transmission has also occurred in some temperate regions of Europe due to the adaptation and spread of vectors (62, 63).

In 2014, the number of chikungunya cases in the Americas increased significantly. Take Puerto Rico as an example: there were 48 local cases in the 27th week (July) of 2014, which rose to 2,305 cases by the 40th week (October) (64). It is reported that there were 909 suspected chikungunya deaths in 22 out of 27 states in Brazil from January 2015 to June 2023. Between the end of 2015 and the middle of 2016, through convergent evolution, strains with the E2-H242 substitution mutation in the East-Central South Africa (ECSA) lineage of chikungunya virus emerged in two independent ECSA lineages in the northern and northeastern areas of Brazil (65, 66). In 2023, the number of reported chikungunya cases in the Americas exceeded 410,000, reaching the highest level in recent years. Approximately 15 countries report chikungunya cases each year. France reported that 118 autochthonous chikungunya cases occurred on Réunion Island from September to December 2024, and as of 16 March 2025, more than 13,000 cases had been reported since the outbreak began. In Mayotte, after two imported chikungunya cases from Réunion Island were reported in 2025, the Mayotte Regional Health Agency confirmed the first autochthonous chikungunya case on the island (67). By the time of 24:00 on September 6, 2025, 5,563 chikungunya cases had been reported in Guangdong Province, China, according to the Guangdong Provincial Center for Disease Control and Prevention (Guangdong CDC) (68). The disease control and prevention departments should strengthen the screening of inbound personnel and the monitoring of local epidemic situations, share information on virus mutations, guide key regions in conducting disinfection and sterilization work, and at the same time popularize among the public the knowledge that “those with fever accompanied by joint pain should seek medical attention in a timely manner,” so as to form a joint prevention and control force with linkage among individuals, institutions and regions.

The West Nile virus (WNV) has shifted from scattered distribution to widespread distribution over the past few decades. Before 2010, its distribution was limited. It was first identified in Uganda in 1937, and spread to a small number of countries such as Egypt, Israel, and France in the 1950s–1960s, with sporadic cases reported in birds, mosquitoes, and horses. In the 1990s, regular outbreaks occurred in the Mediterranean Basin, and large-scale epidemics broke out in Romania and Volgograd (Russia). However, the affected range remained narrow, with genetic lineage differences among countries and no extensive epidemic network (69).

**Figure 2:**
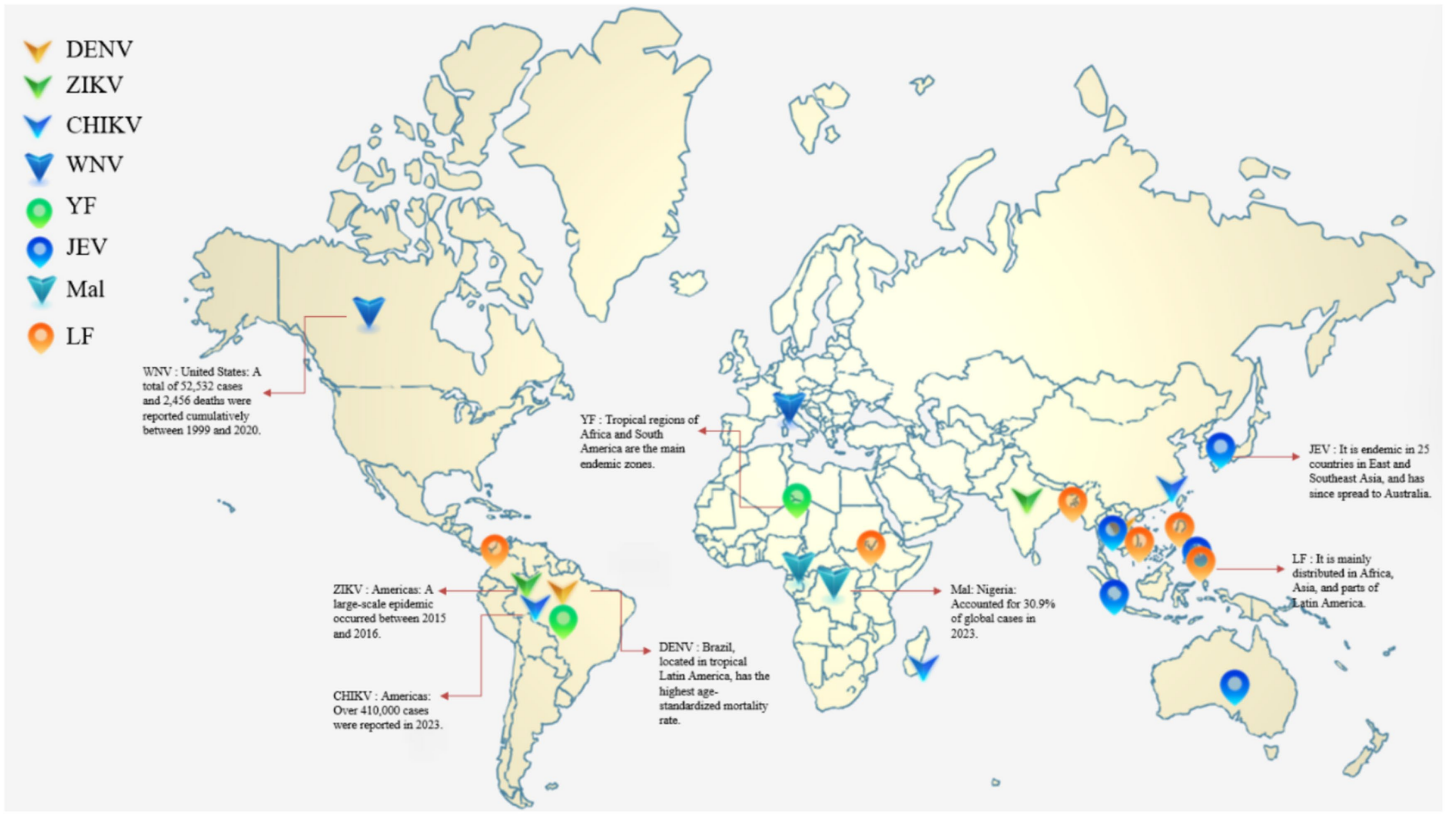
Schematic diagram of the global distribution and epidemiological characteristics of major mosquito-borne diseases. Core data are comprehensively sourced from the WHO Global Health Region standards and relevant articles on PubMed. For ease of understanding, only some key high-incidence areas are marked in the figure, and not all endemic areas are included: ① Dengue (DEN): Thailand recorded the fastest growth in age-standardized incidence rate in East Asia in 2019; Brazil had the highest age-standardized mortality rate in tropical Latin America in 2019. ② Zika virus disease (ZIKV): A large-scale pandemic occurred in the Americas from 2015 to 2016; India experienced case peaks in 2018 and 2021, respectively. ③ Chikungunya fever (CHIK): More than 410,000 cases were reported in the Americas in 2023; over 13,000 cases were recorded on Réunion Island (France) from September 2024 to March 2025; as of September 6, 2025, a total of 5,563 cumulative cases were reported in Guangdong Province, China. ④ West Nile virus disease (WNV): A total of 52,532 cases and 2,456 deaths were cumulatively reported in the United States from 1999 to 2020; as of August 2025, 274 local cases were reported in Italy. ⑤ Yellow fever (YF): Prevalent in tropical and subtropical regions, with tropical areas of Africa and South America serving as the main endemic zones. ⑥ Japanese encephalitis (JE): Endemic in 25 countries in East and Southeast Asia, including rural areas of Japan, Thailand, the Philippines, and Indonesia, and has since spread to Australia. ⑦ Malaria (MAL): Nigeria accounted for 30.9% of global cases in 2023; the Democratic Republic of the Congo accounted for 11.3% of global cases in 2023. ⑧ Lymphatic filariasis (LF): Wuchereria bancrofti is mainly distributed in parts of Africa, Asia, and Latin America; Brugia malayi is predominantly endemic in South and Southeast Asia; Brugia timori is only endemic on certain islands of Indonesia.

After 2010, the virus spread significantly. From 2010 to 2023, 19 Mediterranean countries reported 5,765 human infection cases. Italy (2,280 cases) and Greece were core affected regions, with the epidemic peaking between 2018 and 2022. There were also recorded cases Israel (854 cases) and Tunisia. The virus is transmitted by wild birds, horses, and *Culex pipiens* mosquitoes, with WNV lineages 1 and 2 co-circulating (lineage 2 is dominant in Southern Europe). In 2014, Austria, Hungary, Serbia, and other countries reported local cases, which were concentrated in northeastern Greece and central Hungary. Affected by climate change, the virus is expected to spread to eastern Croatia and northwestern Turkey by 2025, and the number of affected regions will increase to 405 by 2050. In terms of timeline, WNV lineage 1 originated in northwestern Africa in the 20th century, spread to eastern and western Africa in the mid-to-late 1940s, and was introduced from Morocco (North Africa) to Spain (Europe) in the 1980s–1990s, before further spreading to Italy and France. Reverse transmission from Europe to Africa also occurred. WNV lineage 2 originated in South Africa in the 18th–19th centuries, spread to multiple African countries in the 20th century, and was introduced from southern Africa to Hungary (Europe) in the early 21st century. After 2006, it spread to many European countries and became an endemic virus. In 2022, these two lineages caused 965 human infections and 115 deaths in Europe. By August 13, 2025, 8 European countries had reported 335 local infection cases, with Italy being the most severely affected area (274 cases; Figure 2) (69–71).

### 3.2 Socioeconomic impact

The Aedes mosquitoes and the arboviral diseases they transmit (in 2022 US dollars) had cost $94.7 billion cumulatively from 1975 to 2020. When the cost of sequelae is included in the conservative calculation, the total cost is estimated to reach $318 billion. Dengue caused a cost of $76.5 billion between 1975 and 2020, with peaking at $17.5 billion in 2013. Over the same period (1975–2020), the cumulative healthcare costs, including direct medical expenses and direct non-medical expenses, amounted to $46.3 billion, with the highest value of $5 billion in 2016. In the same period, indirect costs totaled $20.7 billion, with peaking at $4.6 billion in 2013; while losses added up to $9.4 billion, with the highest value of $2.5 billion in 2016. In 2017, the cost caused by Zika virus was $2.8 billion. Between 2015 and 2017, the total costs related to Zika virus were estimated at $4.2 billion, averaging $1.4 billion per year. From 2003 to 2020, the reported cost of chikungunya was $9.3 billion, accounting for 10.7% of the total cost, with the highest value of $2.8 billion in 2013. Between 2013 and 2015, the cost caused by chikungunya sequelae was estimated at $219.3 billion, averaging $73.1 billion per year, and the global average cost per case was $2,700 (72, 73). Before 2010, more than 3 million people in New York, the United States, were infected with West Nile virus (WNV). For patients with acute flaccid paralysis (AFP), a complication of WNV, the median initial treatment cost was $25,117 and the median long-term treatment cost was $22,628. The median initial treatment cost for patients with encephalitis was $20,105, while the median long-term treatment cost for patients with meningitis was $10,556. The total cumulative cost of hospitalized WNV patients in the United States from 1999 to 2012 was $778 million (74) (Table 1).

**Table 1:** Systematically summarizes the core information of 8 mosquito-borne diseases, covering disease categories, specific disease types, pathogenic agents, main transmission vectors, and global distribution characteristics.

Disease category	Disease	Pathogenic pathogen	Main transmission vector	Global distribution characteristics
Viral disease	Dengue Fever	Dengue virus	*Aedes aegypti*	Concentrated in tropical and subtropical regions
Viral disease	Zika Virus Disease	Zika virus	*Aedes albopictus*	The core regions are Latin America/Caribbean, Southeast Asia, and Africa
Viral disease	Chikungunya	Chikungunya virus	*Aedes albopictus*	There was local transmission in Europe and Guangdong, China in 2025
Viral disease	West Nile Fever	West Nile Virus	*Culex pipiens*/quinquefasciatus	Europe (Italy, Greece) and the United States are the core regions
Viral disease	Yellow Fever	Yellow Fever Virus	*Aedes aegypti*	Tropical Africa and South America are endemic areas
Viral disease	Japanese Encephalitis	Japanese Encephalitis Virus	Culex tritaeniorhynchus	It is prevalent in 25 countries in East Asia and Southeast Asia, as well as in Australia and China
Parasitic disease	Malaria	Plasmodium	*Anopheles gambiae*/stephensi	Nigeria and the Democratic Republic of the Congo bear the heaviest burden
Parasitic disease	Lymphatic Filariasis	Wuchereria bancrofti	Culex, Anopheles, Aedes	Africa, Asia, and Latin America are epidemic areas

It clearly presents the “pathogen-vector-geographic distribution” correlation logic of mosquito-borne diseases, providing an intuitive reference for understanding the epidemic pattern and prevention/control targets of these diseases.

## 4 Transmission dynamics and drivers

### 4.1 Environmental factors

Studies have shown that different strains of *Aedes albopictus* (Asian tiger mosquito) exhibit variations in temperature adaptation: tropical strains have lower cold tolerance than subtropical and temperate strains. When exposed to fluctuating temperatures below 10 °C for 30 days, tropical strains cease hatching, whereas the latter two strains can hatch under all tested temperature conditions. Furthermore, only subtropical and temperate strains can produce diapausing eggs to overwinter, while tropical strains reproduce year-round. The origin of the strain influences cold hardiness, with subtropical and temperate strains demonstrating significantly stronger cold resistance. Meanwhile, temperature is crucial for the immature stages (larval and pupal stages) of mosquitoes. Taking *Aedes aegypti* (yellow fever mosquito) as an example, the survival rate of its immature stages decreases when temperatures are below 16 °C or above 38 °C, with 26 °C being the optimal temperature. Increased temperatures shorten the development time of immature stages, and temperature stress can also affect the fecundity, survival rate, and body size of adult mosquitoes (75, 76).

Culex has a close relationship with temperature. Firstly, *Culex* mosquitoes are ectothermic organisms, and all stages of their life cycle are significantly affected by temperature. Secondly, although different *Culex* species exhibit differently in their responses to temperature, the development rate and lifespan of *Culex* mosquitoes show a linear relationship with temperature, while survival rate and egg viability display a non-linear relationship (77, 78). Each species has distinct optimal temperature ranges and critical temperatures. For instance, the development time of *Culex pipiens* is the shortest (2.3–10 days) at 30–32.5 °C; at 28–32.5 °C, its lifespan is shorter than the extrinsic incubation period of the West Nile Virus, making virus transmission impossible. The minimum developmental threshold of *Culex quinquefasciatus* ranges from 11 to 18 °C, and the maximum developmental threshold ranges from 34 to 38 °C. Its average lifespan is 32.7 days, which shortens as temperature rises. *Culex* has the shortest development time and the longest lifespan at 25 °C. The development time of the immature stages of *Culex* is negatively correlated with temperature, and the mortality rate of its larvae is high when the temperature is lower than 12 °C or higher than 32 °C (77).

As the primary vectors of malaria, Anopheles are closely related with temperature. Their distribution and activities are restricted by temperature—they mainly inhabit in tropical and subtropical regions, while some species can survive at higher latitudes. Temperature influences malaria transmission by affecting the physiological traits of Anopheles (such as biting rate and development rate) and the extrinsic incubation period of malaria pathogens. Before reaching the optimal temperature, these traits increase almost exponentially with rising temperature, and decline once the temperature exceeds the optimal range. The optimal temperature for year-round malaria transmission is 20.9–34.2 °C. Under future climate warming, the length of the malaria transmission season may increase in areas with temperatures below 25 °C, while it may decrease in areas with temperatures above 35 °C (79, 80). Meanwhile, climate warming may enable Anopheles species more adaptable to warm environments (e.g., *Anopheles stephensi*) to invade and replace existing populations (e.g., *Anopheles gambiae*). The microclimate in urban areas may be beneficial to the development and survival of Anopheles mosquitoes, and the synergy between urbanization and warming may increase the risk of urban malaria transmission, and even promote a shift in disease transmission from malaria to dengue (80).

Humidity is often overlooked, and even when related factors are considered, it is frequently treated as independent of temperature—despite the fact that dry conditions at high temperatures are likely to degrade organismal performance. Spatial and temporal variations in atmospheric humidity affect mosquitoes’ ability to regulate water balance. Maintaining water balance is critical for mosquitoes, as both dehydration and overhydration can negatively impact their health (81). Studies have shown that increased relative humidity generally has positive effects upon mosquito survival, drought tolerance, oviposition and growth, and performance (up to a relative humidity of 90%) (82). Humidity also takes an important part in temperatures, and changes in relative humidity can influence the growth of mosquito-borne pathogens and parasites, as well as mosquitoes’ susceptibility to infection. Temperature and humidity work together to form characteristics of mosquitoes and pathogens. For a given atmospheric humidity, elevated temperatures lead to higher saturation vapor pressure, lower relative humidity, and increased vapor pressure deficit, thereby altering the degree of water stress experienced by mosquitoes. This interaction is important throughout the mosquito life cycle, with a more pronounced impact particularly when temperatures approach the thermal upper limits for certain traits (1).

Rainfall takes a crucial part in building and maintaining moist production sites for mosquitoes, as well as promoting their development and population growth. Moderate rainfall can form breeding grounds, increasing the risk of mosquito-borne disease transmission. However, breeding sites may be destroyed and eggs and larvae washed away by extreme rainfall, thereby reducing the transmission risk (83). Nevertheless, it may also increase people’s exposure to diseases by disrupting private and public mosquito control methods. Heavy rainfall following a drought can fill containers, which then become major breeding sites for mosquitoes. Floods, on the other hand, can drive human hosts away, set up reservoirs for pathogen, and reconstruct breeding habitats for mosquitoes. Generally, the effect of precipitation depends on the season in which it occurs, with the greatest influence in the early stage of the transmission season and the least in the later stage (84).

### 4.2 Ecological drivers

#### 4.2.1 Urban ecological driving factors

Various infrastructures and objects in urban spaces provide suitable artificial breeding grounds for mosquitoes. In particular, small water-holding containers generated by buildings, construction sites, etc., are highly prone to mosquito breeding, making *Aedes aegypti*, *Aedes albopictus* and other species more adaptable to artificial environments. Although urban green infrastructure is continuously integrated, the difficulty in managing stagnant water also makes it a potential habitat and breeding ground for mosquitoes (85). The urban heat island effect raises the surface temperature of cities, which is usually 2–3 °C higher than that of surrounding rural areas. As ectothermic animals, mosquitoes have their physiological functions, ecology and dispersal significantly affected by temperature: increased temperature shortens the development time of larvae, while increasing the mortality rate of both larvae and adults, thus exerting a balancing effect on mosquito reproduction. Temperature also influences mosquito behaviors such as flight activity, biting rate and reproduction. A higher metabolic rate can extend flight time and has a significant impact on the biting rate (86). Informal settlements have a unique thermal environment and lack heat mitigation measures, which may amplify the impact of temperature on disease vectors. Rising temperatures caused by the intensification of global climate change and urban heat island effect may increase the population of *Aedes aegypti* and exacerbate heat stress. Informal settlement upgrading projects may intensify the urban heat island effect and increase the threat of dengue fever because of the increase of impermeable surfaces and building structures, while nature-based solutions such as retaining green spaces can alleviate these impacts (87).

#### 4.2.2 Agro-ecological and wildlife-related driving factors

In the “human-animal-environment” interaction system, agricultural practices, land use changes, and wildlife hosts collectively shape the transmission pattern of mosquito-borne diseases through multi-dimensional effects, with close synergistic or restrictive relationships existing between these factors. In agricultural activities, large-scale irrigation projects and differences in farming methods have a particularly significant impact on mosquito habitats. A study in western Ethiopia shows that the annual entomological inoculation rate (EIR) of *Anopheles arabiensis*—the malaria vector—in large-scale sugarcane irrigation areas (102 infective bites per person per year) is 5.7 times that in non-irrigated areas (18 infective bites per person per year). Its average human-biting rate and sporozoite rate are also 2 times and 2.3 times those in traditional irrigation areas, respectively. Stable irrigation water bodies enable mosquitoes to reproduce year-round, breaking the seasonal constraints on disease transmission. In contrast, in northern Benin, after rice paddies adopted the “reduced tillage + intermittent flooding” model, the density of Anopheles larvae decreased by 80.8, 30.8, and 40.7% during the rice transplanting stage, tillering stage, and maturity stage, respectively, compared with the traditional “deep tillage + continuous flooding” model. This approach not only reduces mosquito breeding sites but also achieves efficient water use, providing a feasible path for the synergy between agricultural production and disease prevention and control (88).

Land use changes further exacerbate or mitigate transmission risks through spatial restructuring. Deforestation in Borneo, Malaysia, has caused long-tailed macaques—natural hosts of *Plasmodium knowlesi*—to migrate to human farmlands and villages. From 2015 to 2017 in Sabah, Malaysia, 80% of patients infected with Plasmodium knowlesi lived within 5 kilometers of non-human primate habitats. Additionally, the rate of farmland expansion in these areas was 1.5 times that of other regions, which significantly increased the spatial overlap between wildlife and humans. In Ontario, Canada, predictions using the Dyna-Clue model indicate that by 2070, deforestation in the midwestern region will increase the habitat area of *Culex pipiens-restuans*—the vector of West Nile virus—by 146.9% compared to 2020. Increased water exposure and rising temperatures caused by forest loss will accelerate mosquito development and population expansion. During the urbanization process in Europe, the urban heat island effect in urban fringe areas has extended the active period of mosquito vectors by 2 to 3 months. Models predict that by 2050, the probability of West Nile virus infection in these regions will increase by 30 to 50% compared to the current level. The increase in artificial water bodies (such as waterlogged sewers and discarded containers) has also provided dense breeding sites for mosquito vectors (69, 89, 90).

As natural reservoirs of mosquito-borne pathogens, wildlife directly determine the maintenance and cross-species transmission of pathogens through their population dynamics and host roles. Birds are core amplification hosts for West Nile virus. Studies in the European Mediterranean Basin have found that the virus infection rate in corvid birds is positively correlated with the proportion of farmland and urban fringe land. During the 2020 West Nile virus outbreak in Spain, approximately 30% of the virus genotypes were highly homologous to the strains carried by migratory birds from North Africa, making bird migration a key vector for the geographical spread of the virus. For JEV, there exists a wild cycle of “wading birds—mosquito vectors” and a domestic cycle of “pigs—mosquito vectors.” In Asia, the expansion of paddy field areas (frequently used by wading birds) and the development of the pig industry provide sufficient resources for vector mosquitoes such as *Culex tritaeniorhynchus*. Additionally, for every 1-kilometer reduction in the distance between paddy fields and pigsties, the human infection rate increases by 15 to 20%. The interaction between the two cycles significantly enhances the efficiency of virus transmission (91, 92).

In addition, the spatial planning of irrigated agriculture must also incorporate considerations of transmission risks. A study in Malawi, which analyzed the impact of irrigation development on suitable mosquito habitats, found that land use/cover changes caused by irrigation expanded the maximum suitable breeding area of *Anopheles gambiae s.s*. from 8.60 to 15.16% during the rainy season and from 1.78 to 2.17% during the dry season. Targeted water resource management, such as ditch dredging and irrigation scheduling, can effectively reduce suitable mosquito habitats, providing a basis for the spatial synergy between irrigation projects and disease prevention and control (93). From 2010 to 2023, the epidemic intensity of West Nile virus in the Mediterranean Basin showed a significant correlation with land use types (such as the proportion of farmland and wetlands), bird migration routes, and climatic conditions (temperature and precipitation).

**Figure 3:**
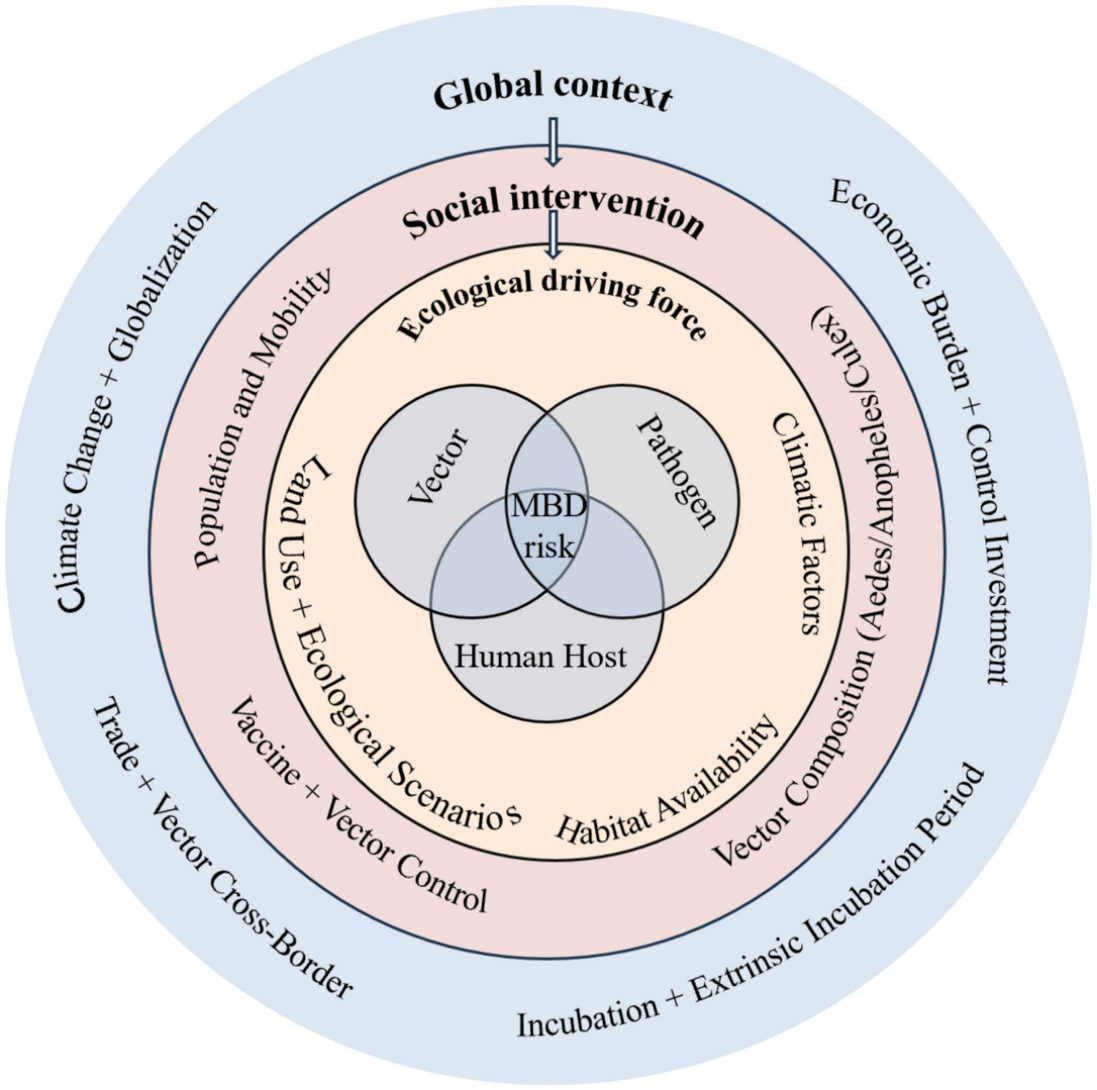
Intuitively presents a multi-dimensional driving framework for mosquito-borne disease (MBD) risk, decomposing the core influencing factors of disease transmission and intervention pathways to provide a systematic perspective for understanding its epidemiological mechanism. Centered on “MBD risk,” the framework covers four major driving factors: global context (including economic burden, prevention and control investment, social intervention, climate change, globalization, and population mobility), ecological drivers (pathogen characteristics, climatic conditions such as temperature and humidity, and accessibility of vector habitats), human-related dynamic processes (trade and cross-border vector transmission), and clarifies the core role of intervention measures such as vaccination and vector control. The schematic diagram integrates the core discussions on environmental factors, ecological drivers, socioeconomic impacts, and prevention and control strategies in the article, intuitively demonstrating the interaction of elements in the “human-animal-environment” system and providing logical support for the subsequent formulation of targeted prevention and control measures.

This further verifies the comprehensive role of various elements in the “human-animal-environment” system in the transmission of mosquito-borne diseases (71) (Figure 3).

### 4.3 Critical interactions between core drivers

The complexity of mosquito-borne disease transmission arises from the interdependencies among core driving factors, which also constitutes a key rationale for formulating targeted strategies. Climate change and urbanization collectively drive temperature elevation, which not only accelerates the development of mosquito larvae but also synergizes with the urban heat island effect to expand the distribution range of vectors. These interconnections highlight the limitations of single-factor analysis; instead, the “climate-urban-agriculture” synergistic effect can serve as a critical target for intervention measures.

## 5 Prevention and control strategies

### 5.1 Vector control measures

Currently, commonly adopted intervention strategies include using insecticides.

They also include eliminating mosquito breeding sites. Moreover, conducting public awareness campaigns to enhance people’s understanding of methods for preventing mosquito bites is another strategy. Using insecticide-treated bed nets is also among these strategies. Finally, collecting and analyzing data through monitoring, detection, research, and development to further understand and predict these diseases and their consequences is an important part of the intervention strategies (94). Pyrethroids are the most widely used insecticides for IRS. Currently, they are also the only synthetic insecticides used in ITNs and fabrics. However, the prevalence of mosquito resistance to pyrethroids has become a major problem in combating disease transmission across the globe. In Africa specifically, the proportion of Anopheles mosquitoes that are resistant to pyrethroids is as high as 78% (95).

Most existing intervention measures adopt a “superimposition of individual strategies” model rather than a “systematic synergy” model. For instance, after large-scale distribution of Insecticide-Treated Mosquito Nets (ITNs) in a certain area, no concurrent community campaigns to “eliminate domestic stagnant water (and reduce mosquito breeding)” were launched. This resulted in excessively high mosquito density, which offset the effectiveness of ITNs and reduced their usage efficiency.

Environmental management strategies serve to cut down mosquito larval habitats, like turning garbage zones into vegetable plots, converting barren lands into parking areas, fixing damaged water pipelines, sealing cellars, and building wastewater drainage systems. Such actions can essentially alter the breeding surroundings favorable to mosquitoes (96). Temporary adjustments are applied to mosquito habitats, and these adjustments involve managing necessary container s—for instance, fitting wooden lids or mesh frames on water storage vessels, clearing basement leaks, handling buckets and flowerpots, and cleaning drum containers and dish racks—to diminish mosquito breeding spots (97).

In existing data monitoring, most efforts only focus on “insecticide resistance rates” and fail to link to key information such as “local pesticide application frequency” and “types of environmental breeding sites.” This situation cannot provide a precise basis for strategy adjustments, and ultimately traps intervention work in a dilemma of “disconnection between monitoring and action.” It is possible to integrate various vector control-related measures, including the potential use of insecticides (e.g., pyrethroids) and environmental management (e.g., eliminating stagnant water to reduce vector breeding sites). At the same time, incorporate comprehensive surveillance of vectors and diseases (such as vector density monitoring, insecticide resistance monitoring, and case tracking) to ensure that control measures are supported by data. Meanwhile, the integration of comprehensive surveillance for vector organisms and diseases (such as vector density monitoring, insecticide resistance monitoring, and case tracking) ensures that prevention and control measures are supported by data, which bears certain similarities to the concept of Integrated Vector Management (IVM).

Integrated Vector Management (IVM) is derived from Integrated Pest Management (IPM), with “prevention first, integration of multiple tools, and data-driven approaches” as its core principles; it focuses on reducing the burden of vector-borne diseases and supports the goal of “One Health” (the health of humans, animals, and the environment), and its decision-making is centered on “health thresholds” (such as vector-borne disease risks and the number of infected cases), which differs from IPM that is guided by economic thresholds. IVM comprises four core strategies, namely cultural control (including community education, hygiene management, and personal protection), physical/mechanical control (such as environmental modification, physical barriers, and landscape management), biological control (like the use of natural enemies, pathogens, and genetic technologies), and chemical control (covering insecticides and other substances, which requires data support and prioritizes low-risk formulations); among these, insecticides serve only as supplementary tools and are used solely when vector density exceeds the threshold or cases occur, while risks must be constrained through resistance monitoring and precise application, and dependence on them should be reduced by relying on non-chemical strategies. On the whole, IVM emphasizes community participation, ecological protection, and multi-strategy synergy, rather than relying solely on chemical means (98).

### 5.2 Vaccination strategies

Vaccines, as a key means of preventing and controlling mosquito-borne diseases, have made significant and undeniable progress in the research and development of vaccines against emerging and re-emerging flaviviruses. Specifically, the research and development of vaccines for various mosquito-borne diseases present different situations (37):

Dengue vaccines: At present, there are only two commercially available dengue vaccines worldwide. Dengvaxia, one of the earlier vaccines to enter the market, is mainly suitable for people with a history of dengue fever. However, it has certain limitations: it is less effective in children and dengue patients, and makes people who have not been infected with dengue more likely to face the risk of severe dengue infection (99). TAK-003, approved by the World Health Organization in 2023, is specifically for kids from 6 to16 years old in highly endemic regions. It showed good immunogenicity in clinical trials and can effectively reduce the incidence of severe cases (100). In addition to the two commercialized tetravalent dengue vaccines, Dengvaxia and TAK-003, a number of vaccines are in different stages of research and development: TV003/TV005 are tetravalent live-attenuated vaccines, attenuated via gene deletion; Phase III trials in Brazil showed their protection rates reached 80 and 89%, respectively, for populations with no prior dengue infection and those with a history of infection, and they can take effect with a single dose. TDEV-PIV is a tetravalent inactivated vaccine that requires adjuvants to enhance immunity; Phase I trials in the United States confirmed it can induce long-lasting immune responses, with protective efficacy lasting up to 3 years. V180 (DEN-80E) is a recombinant subunit vaccine containing truncated envelope proteins of 4 serotypes; Phase III trials demonstrated a 79.6% effectiveness rate in preventing dengue with no reports of severe cases, though it requires multiple booster immunizations and its safety still needs verification. Additionally, DNA vaccines (such as TVDV) are in the animal and Phase I evaluation stages; virus-like particle (VLP) vaccines have shown good results in non-human primate studies but face challenges in particle assembly and immunogenicity optimization; while mRNA vaccines have made progress, they have issues where E protein mutations cannot effectively reduce the antibody-dependent enhancement (ADE) effect (101, 102).

Zika virus vaccines: Researchers have explored a variety of vaccine platforms where inactivated vaccines, live attenuated vaccines, and nucleic acid-based vaccines are included. Some candidate vaccines have shown promising immunogenicity in preclinical studies, which can induce corresponding immune responses in the body (103). However, the path from preclinical research to clinical success is not smooth, and the transformation of different candidate vaccines varies greatly. Immune persistence is a key issue—whether the immune protection induced by the vaccine can last for a long time to protect vaccinated individuals from Zika virus for a long period (19). In traditional platforms, whole-virus inactivated vaccines (ZPIV, TAK426), DNA vaccines (VRC5283, GLS5700), adenovirus-vectored vaccines (Ad26. ZIKV.001), and mRNA vaccines (mRNA-1893) are mostly in Phase 1 clinical trials. They are proven to be safe and immunogenic, but some face issues such as insufficient immune persistence or the need for booster doses (104).

Chikungunya virus vaccines: Two candidate vaccines, IXCHIQ and PXVX0317, have achieved remarkable results in the research and development process of chikungunya virus vaccines and have completed the clinical development stage (25). IXCHIQ performed excellently in phase 3 trials, showing strong immunogenicity and effectiveness, and has been approved for use in some regions. It uses live attenuated virus technology and triggers immune responses through virus strains from East, Central, and South Africa, but there are also certain adverse reactions—for example, its use in people aged 60 and above has been suspended (105). PXVX0317 showed promising results in early trials, but further research and evaluation in a larger population are needed to confirm its safety and effectiveness. Only after full verification can this vaccine be promoted more widely, providing stronger support to prevent and control chikungunya fever.

Malaria vaccines: The R21 malaria vaccine has attracted much attention due to its excellent clinical efficacy. Given the high incidence of malaria in Africa, the future production of the vaccine is likely to exceed the demand in Africa, which will bring new hope to malaria prevention and control in Africa and even globally. A large number of clinical trial data reveal that the R21 malaria vaccine is efficient in preventing malaria, which can effectively reduce the incidence and mortality of malaria, providing an important new tool for global anti-malaria efforts (106). Additionally, Mosquirix, as the world’s first malaria vaccine, was recommended by the World Health Organization (WHO) in 2021 for use in children in regions with moderate to high transmission of *Plasmodium falciparum* malaria, such as sub-Saharan Africa. Phase III clinical trials showed that it can reduce the clinical incidence of malaria by 36% in children aged 5–17 months and by 25.9% in infants aged 6–12 weeks, with an effective rate of approximately 30% in preventing severe malaria in children. Data from large-scale pilot programs indicate that the vaccine has a good safety profile, with only mild adverse reactions reported (107, 108).

To date, no West Nile Virus (WNV) vaccine for humans has advanced beyond Phase I/II clinical trials. While a variety of candidate vaccines (such as live attenuated chimeric vaccines and DNA vaccines) have demonstrated certain potential, their development is still restricted by issues including immunogenicity and large-scale production. Four WNV vaccines for horses have been approved and have shown good efficacy. Meanwhile, the approved dengue vaccines have provided references for the development of WNV vaccines. Most antiviral drugs targeting WNV-related proteins are still in the *in vitro* experimental stage, and more *in vivo* studies are needed to promote their practical application (31).

These vaccines still face numerous challenges, including the difficulty of cross-protection, where differences between various virus strains make it hard for vaccines to provide comprehensive and effective protection against all strains; safety issues, as some vaccines may trigger adverse reactions in recipients, such as allergic reactions and immune system abnormalities. Particularly for certain special groups, like children, women who are pregnant, and individuals who are weak in immune systems, the safety of vaccines requires rigorous evaluation and assurance. Additionally, the production process is lengthy and often consumes a great deal of time and resources, which underscores the urgent need for innovative vaccine approaches.

### 5.3 Chemoprophylaxis and treatment

The treatment regimen for uncomplicated malaria should be determined based on the type of Plasmodium infection and drug resistance patterns. For *Plasmodium falciparum*, *Plasmodium knowlesi*, and chloroquine-resistant *Plasmodium vivax*, artemisinin-based combination therapies (ACTs) are recommended, including artemether-lumefantrine and artesunate-mefloquine. Among these, artemether-lumefantrine should be taken with food to optimize absorption (109). Chloroquine is the preferred option for the treatment of chloroquine-sensitive *Plasmodium vivax*, *Plasmodium malariae*, and *Plasmodium ovale*.

In cases of severe malaria, regardless of the Plasmodium species involved or the patient’s pregnancy status, intravenous artesunate should be administered immediately (110). Individuals traveling to malaria-endemic areas need to take chemoprophylactic drugs. The selection of drugs depends on multiple factors, such as whether malaria in the area is chloroquine-sensitive, potential adverse reactions of the drugs, administration frequency, and patient preferences. The RTS, S/AS01 malaria vaccine was approved by the WHO for global use in children in malaria-endemic countries in October 2021. This vaccine has a 46% protective efficacy against clinical malaria in African children within 18 months, and a 34% protective efficacy against severe malaria, malaria-related hospitalization, and all-cause hospitalization (111).

For viral mosquito-borne diseases, treatment primarily consists of supportive care. Currently, there are no specific antiviral drugs available for dengue fever, Zika virus, chikungunya fever, or West Nile virus, although several candidate drugs are under development. Antiviral strategies targeting viral entry, replication, and assembly have demonstrated activity in preclinical models but are still in the exploratory stage.

### 5.4 Novel control technologies

In addition to the application of plant-derived insecticides mentioned (112), new methods for reducing mosquito populations include the following aspects.

#### 5.4.1 Larvicidal proteins of *Bacillus sphaericus*

The binary larvicidal proteins (BinA/BinB) of *Bacillus sphaericus* are the core of the bacterium’s larvicidal activity. They function by binding to the GPI-anchored protein Cqm1 in the midgut of Culex mosquitoes and internalizing through a pore-forming mechanism. These proteins are effective against Culex and Anopheles larvae but inactive against *Aedes aegypti* larvae. Additionally, it is mentioned that chemical modifications (such as pegylation of BinA) and chimeric protein construction can optimize their activity and address resistance issues (113). For the *Bacillus sphaericus* strain Q001, cation exchange chromatography and guanidine hydrochloride extraction methods were optimized to obtain its surface layer (S-layer) proteins. Through SDS-PAGE and MALDI-TOF mass spectrometry, a 120 kDa S-layer protein was identified. Bioassays showed that the LC₅₀ of this protein against *Aedes aegypti* larvae was 11 μg/mL, and it also exhibited toxicity to Culex and Anopheles larvae, indicating its potential to replace chemical larvicides (114).

#### 5.4.2 Wolbachia-based symbiotic control technology

The development of Wolbachia in the early stage, the focus was on technology implementation: leveraging the differences in weight, morphology, and emergence time between male and female pupae of *Aedes aegypti* infected with this bacterium, a mechanical glass separator was used to achieve the screening of male mosquitoes with a purity of over 99%. Combined with 70 Gy radiation, this method rendered mosquitoes completely sterile while ensuring the field competitiveness of male mosquitoes, solving the core problem of the combined use of “SIT + IIT” and laying the foundation for large-scale application (115). Subsequently, the research shifted toward quantitative studies. Through a pulsed differential equation model, the synergistic mechanism between its Cytoplasmic Incompatibility (CI) effect and the Sterile Insect Technique (SIT) was clarified, and the critical threshold for releasing sterile male mosquitoes was calculated. Two control strategies—"open-loop (fixed cycle)” and “closed-loop (dynamic adjustment based on population density)”—were proposed along with quantitative schemes (e.g., 10,675 mosquitoes per hectare for a 7-day release cycle), promoting mosquito control from an empirical approach to a precise one (116). The latest research has delved into the molecular level, confirming that its surface protein (WSP) can bind to the host’s STK (a dengue virus-promoting protein), thereby reducing the latter’s availability to the dengue virus. Additionally, it regulates STK bidirectionally—either for its own colonization or antiviral purposes. This reveals the antiviral pathway from the perspective of protein interaction, enhances the mechanistic support, and drives the technology to leap toward a mature system (117). Thus, the development of *Wolbachia* in mosquito control has gradually deepened along the path of “practical optimization → theoretical quantification → mechanism analysis.”

## 6 Conclusion

In summary, the key contributions of this review are reflected in three aspects: (1) systematically clarifying the synergistic effects of environmental, ecological, and socioeconomic factors on the transmission of mosquito-borne diseases (e.g., the interactions between temperature-humidity-mosquitoes-pathogens); (2) constructing a “driving factors-preventive and control measures” priority framework for different epidemic scenarios; (3) identifying emerging technologies (such as Wolbachia technology and the R21 vaccine) as critical breakthrough directions to address existing challenges (insecticide resistance, shortages of certain vaccines). Future research should further explore the molecular mechanisms underlying the interactions between driving factors and pathogens (e.g., how urban thermal environments affect the immune response of Aedes mosquitoes to viruses) to refine targeted strategies.

## Methods

We searched three core academic databases, namely PubMed, Web of Science, and Scopus. Meanwhile, we adopted surveillance reports and prevention/control guidelines released by authoritative institutions such as the World Health Organization (WHO), the European Centre for Disease Prevention and Control (ECDC), and the Guangdong Provincial Center for Disease Control and Prevention of China.

Peer-reviewed original studies, systematic reviews, and official reports with clear data sources were included. Priority was given to literatures focusing on the incidence rate, mortality rate, vaccine development, and prevention/control effects of mosquito-borne diseases, with a sample size of ≥100 cases or rigorous study designs (e.g., randomized controlled trials, long-term cohort studies), with duplicated published literatures and non-English academic literatures excluded.

Two researchers independently conducted the initial screening (based on titles and abstracts) and secondary screening (full-text verification). Disagreements during the screening process were resolved through joint discussion. In the secondary screening stage, the reliability of literature data was verified with emphasis. For institutional reports and the latest epidemic information (e.g., data related to chikungunya and West Nile virus infection from 2024 to 2025), the accuracy of information was ensured by cross-verifying with official notifications.
